# State of the maternal healthcare continuum in Guinea, awaiting the next Demographic and Health Survey: the case of the five communes of Conakry in 2022

**DOI:** 10.3389/frph.2024.1324011

**Published:** 2024-09-12

**Authors:** Niouma Nestor Leno, Daniel William Athanase Leno, Abdoulaye Sow, Gaston Kambadouno, Alioune Camara, Serge Mayaka, Alexandre Delamou

**Affiliations:** ^1^ African Center of Excellence for the Prevention and Control of Transmissible Diseases (CEA-PCMT), Gamal Abdel Nasser University of Conakry, Conakry, Guinea; ^2^ Department of Public Health, Faculty of Health Sciences and Techniques, Gamal Abdel Nasser University of Conakry, Conakry, Guinea; ^3^ Department of Gynecology and Obstetrics, National Donka Hospital, Conakry, Guinea; ^4^ Kinshasa School of Public Health, Kinshasa, Democratic Republic of Congo

**Keywords:** continuum of maternal health care, associated factors, Conakry, Guinea, 2022

## Abstract

**Background:**

The continuum of maternal health care ensures consistency in the delivery of care from pregnancy to the postnatal period. It recommends a minimum of 4 antenatal visits, skilled birth attendance, and 42 days of postnatal care. This approach helps reduce maternal deaths. The aim of this study was to estimate the proportion of women who had completed the different stages of the continuum of maternal health care (four antenatal visits, given birth under the care of qualified personnel, and received postnatal care within 42 days of delivery).

**Methods:**

This was a cross-sectional analytical study conducted in the five communes of Conakry, using a two-stage cluster sampling for data collection. Results were described using medians and percentages. The proportions of women in the continuum of care, and at the different stages of this continuum, have been weighted. Multivariate logistic regression was used to identify the factors associated with non-completion of the different stages of the maternal health care continuum among the women included in this study.

**Results:**

We found that 26.9% of women had completed all stages of the maternal health care continuum, while 73.1% had not. While 56.7% received four antenatal visits, only 29.5% delivered under the care of a qualified healthcare professional. Key factors associated with discontinuity were not attending school (AOR 1.825: 1.594–2.089), unemployment (AOR 4.588: 3.983–5.285), having two or more living children (AOR 1.890: 1.016–1.296), and not receiving a free Long-Lasting Insecticidal Net at the first Antenatal Care.

**Conclusion:**

Maternal care discontinuity is a major issue in Guinea. The country's Health Development Plan had set an expected level for maternal care which has not been met as of 2022. The completeness of care is influenced by various factors, including individual socio-demographic characteristics and factors related to the organization, availability, and quality of health services. To reduce maternal and child mortality rates, it is essential to improve interpersonal communication during antenatal care, ensure the availability of quality health services, and conduct a national study on maternal health service quality and maternal satisfaction. This will help establish a proper continuum of care for mothers and children.

## 1 Introduction

Maternal mortality remains a significant global health issue. There were approximately 810 maternal deaths per day in 2017, with 94% of them occurring in low-income countries (1). Sub-Saharan Africa continues to be the region with the highest number of maternal deaths, accounting for 67% of all such deaths worldwide (2).In countries with limited resources, the maternal mortality rate is as high as 462 per 100,000 live births. By contrast, in high-income countries, this rate is only 11 per 100,000 live births, as per the statistics of 2017 (2). Complications during and after pregnancy are the leading cause of death for women. However, some of these complications may exist before pregnancy and worsen during it if not addressed within the framework of the women's care (3).

Severe bleeding (especially after childbirth), postpartum infections, high blood pressure during pregnancy (pre-eclampsia and eclampsia), and complications related to unsafe abortion are the leading causes of maternal deaths, accounting for 75% of all causes (4). Other infections (malaria, HIV, etc.) and chronic diseases (heart diseases, diabetes, etc.) account for the rest of the causes of maternal deaths (4).

It is known that many maternal deaths could be prevented if appropriate care were available and accessible for the health of the mother, newborn, and child (4, 5).These cares include quality family planning, skilled care during pregnancy, childbirth (6, 7), and after birth, as well as quality services after an abortion and the ability to have a safe abortion when allowed by law.

Efforts made to achieve the Millennium Development Goals (MDGs) have resulted in a significant reduction in maternal deaths. According to the United Nations report published in 2017 on the MDGs for 2015, maternal mortality has decreased by 45% worldwide since 1990, and the under-five mortality rate has been reduced by more than half (5). Despite significant progress, maternal healthcare coverage services remain unequal in low- and middle-income countries (6). Therefore, for implementing actions related to the Sustainable Development Goals (SDGs), improving maternal, neonatal, and child health is strongly recommended (7).

In recent decades, there has been an increased focus on implementing the continuum of care approach to improve the quality of maternal health services. The continuum of maternal health care ensures consistency in the delivery of care from pregnancy to the postnatal period. It recommends a minimum of 4 antenatal visits, skilled birth attendance, and 42 days of postnatal care. This approach helps reduce maternal, neonatal, and child mortality rates (8). The continuum of care is a public health intervention that is simple, cost-effective, and low-tech. Its aim is to address the health challenges of mothers, newborns, and children. It involves providing care throughout different stages of life, including adolescence, pregnancy, childbirth, the postnatal period, and childhood. The goal is to improve the health and survival of both mothers and children (9). A meta-analysis has shown that continuous care before and after pregnancy can reduce the risk of neonatal and perinatal mortality by 21% and 16%, respectively, leading to a good continuum of care that helps reduce the maternal mortality rate (10).

However, the proportion of women benefiting from a continuum of maternal health care is low in resource-limited countries, especially in sub-Saharan Africa. A study conducted in Ghana in 2015 showed that only 8% of women benefited from a good continuum of maternal care (11). Another study conducted in Ethiopia in 2020 revealed that only 12.1% of women had completed the continuum of maternal care (12).

In Guinea, progress has been made in recent years to improve maternal and newborn health indicators. However, further efforts are still needed to save the lives of mothers and children. According to the 2018 Demographic and Health Survey (DHS), 81% of women who had a live birth received antenatal visits care from a qualified provider. However, only 35% of women had attended at least 4 antenatal visits. Additionally, just 55% of births were assisted by qualified health personnel. Furthermore, only 49% of women had a postnatal examination within 48 h of birth (13).A secondary analysis based on 2018 Demographic and Health Survey data showed that only 20% of women benefit from a good continuum of maternal care in Guinea (14). It is worth noting that the latest study on the maternal care continuum in Guinea is one of the few studies available, but it relies on outdated data. For a health program to be effective, regular assessments are essential to identify hindrances and suggest corrective actions promptly, for a better outcome. The current study provides updated data on the continuum of maternal care in Conakry, Guinea. In this study, we did not compare our method to that of DHS. We simply wanted to show the importance of having approximate data on certain key health indicators between two DHS surveys, which often take a considerable amount of time to complete. This allows health actors to adjust intervention approaches promptly to prevent the worsening of a health issue due to delayed identification. This serves as a form of internal self-assessment, awaiting an external evaluation of the impact of health interventions, which is typically conducted through DHS surveys carried out every five years. It is not our intention to compare the DHS with our survey. We simply want to highlight the need to carry out small periodic surveys between two DHSs to assess progress towards national health objectives. This will enable the Ministry of Health to readjust operational interventions prior to the evaluations that are often carried out through the DHS. As we know, DHS surveys are carried out every five years. We believe that waiting five years to undertake certain corrective actions could compromise the achievement of health status results. In short, this survey must guide operational actions. The objective of this study was to estimate the proportion of women who had completed the various stages of the continuum of maternal health care, i.e., those who had completed the four antenatal visits, given birth under the care of qualified personnel, and received postnatal care within 42 days of delivery, during the 12 months preceding the survey in Conakry, as well as to identify the factors associated with non-completion of the various stages of the continuum of maternal care.

## 2 Materials and methods

### 2.1 Study design

This is a cross-sectional analytical study conducted based on prospectively collected data from women who gave birth in the 12 months preceding the survey.

### 2.2 Study setting

This study was carried out in the five communes of the city of Conakry (Dixinn, Kaloum, Matam, Matoto, and Ratoma). Conakry is the political capital of the Republic of Guinea, a West African country covering an area of 245,857 km^2^. The country is divided into four natural regions (Lower Guinea, Middle Guinea, Upper Guinea, and Forested Guinea). Administratively, the country has 8 administrative regions, including the special area of Conakry. Each administrative region is divided into prefectures, corresponding to the health district in terms of health administration. In total, Guinea has 38 health districts, including 5 in Conakry and 33 in the interior of the country.

Based on projections from the National Institute of Statistics, Guinea's population in 2022 was estimated to be 13,261,638 with women accounting for 52% of the population. The annual population growth rate is 2.9%, while the synthetic fertility rate is 4.8 children per woman. Unfortunately, the literacy rate in Guinea remains low, with only 32.0% of individuals aged 15 and older considered literate. The socio-economic situation in Guinea is also characterized by persistent poverty, with 43.7% of the population living below the poverty threshold (15). In addition to the high maternal mortality rate and low healthcare coverage, the country is grappling with the emergence and re-emergence of epidemic diseases (16).

### 2.3 Study population

The research focused on women between the ages of 15 and 49 who had given birth within the 12 months prior to the survey. A total of 5,335 women were interviewed in March 2022 across five communes in the city of Conakry. The reason for selecting women who had given birth within the last 12 months preceding the survey was to minimize any potential errors in maternal recall.

### 2.4 Sampling and data collection

We conducted a study using a two-stage stratified sampling method based on the “World Health Organization” type. The city of Conakry consists of five communes, each of which was considered a stratum. In the first stage, we selected primary units or clusters in each commune. The technical team carried out this selection based on the list of neighborhoods in the communes from the third General Population and Housing Census (RGPH-3) of 2014 in Guinea. The census was updated in 2017 by the National Institute of Statistics and included 9,668 enumeration areas, 1,505,805 households, and 11,555,061 residents in 201 (17). The census district/sector was chosen as the primary unit. In each commune or stratum, 30 units were randomly selected, irrespective of the number of base units contained in the sampling frame of the various communes. The number of units was not identical in all communes. However, each commune had the necessary number of units in the sampling frame to be able to draw the 30 units envisaged by the survey.

The city of Conakry comprises 5 communes. Within each commune, we selected 30 units, amounting to a total of 150 primary units. The sampling frame for the primary units (clusters) used for this study was that established by the National Institute of Statistics after the third general population census (RGPH-3) of 2014 and updated in 2017. The sampling frame for the primary units was the exhaustive list of all enumeration areas in each commune. The selected primary units were also referred to as “clusters” during data collection. Households located within the perimeter of the selected primary unit were considered secondary units.

The process of household sampling was carried out in a randomized manner within the neighborhood. Upon entering the field, the data collection teams conducted a comprehensive reconnaissance of the cluster's environs to identify all boundaries and contours. The selection of households containing the survey targets was done in accordance with the method recommended by the WHO for coverage surveys in households and the investigator's guide developed for this purpose (18).

After the reconnaissance, the data collection agent positioned themselves at one of the corners and threw a pen. The pen's tip indicated the direction to follow. The agent counted two compounds and started data collection in the third compound. They then continued in this manner until they reached the quota of eligible women for the study. In each household, all eligible women were interviewed. To avoid overlaps, clusters belonging to the same neighborhood were assigned to the same agent.

Data were collected using a pre-tested questionnaire designed on the Kocollect application. The questionnaire was administered to target women by medical students in their final year of medical school, trained for this purpose. The language used during the administration was either French or the national language, depending on the understanding of the women. In order to ensure comprehension, the questionnaire was translated into Guinean national languages during the training of the investigators. The information collected for this study was based on self-declarations made by the women during interviews with the investigators.

### 2.5 Definition of study variables

• Dependent Variables

The outcome variable of this study was the continuum of care for maternal health services. The continuum of care is a composite indicator, which was constructed as a binary variable. This means that a woman was considered to have received continuum of care if she reported receiving services at the following three levels:

− At least four antenatal care visits for pregnancy monitoring.
− Delivery assisted by a qualified healthcare professional (doctor, nurse, midwife).
− Postnatal care for the mother and newborn within 42 days or six weeks after childbirth (19).

The continuum of care for women and mothers is considered incomplete if any of the three stages are missed. Continuous care during pregnancy is defined as completing antenatal visits, while continuous care during delivery is defined as delivering with the assistance of qualified personnel and having antenatal visits. Lastly, the completion of four antenatal care visits, delivery by qualified personnel, and postnatal care are considered continuous care at the postpartum level, which is also deemed a complete continuum of care (20).

• Independent Variables

Based on Owilli et al.'s continuum of care, the conceptual model consists of four main components: family and individual, socio-economic, child characteristics, and field (21). The family and individual factors include the mother's age, ethnic origin, religion, and obstetric history. Information on services received during antenatal and postnatal visits was also collected. The socio-economic factors include the mother's level of education and occupation (21).

### 2.6 Data analysis

The data analysis was conducted using IBM SPSS Statistics version 25. To summarize numerical variables, descriptive statistics were employed, presenting the data as medians with their corresponding interquartile ranges or means with their standard deviations. On the other hand, categorical variables were summarized by calculating proportions with their corresponding confidence intervals. The proportions of women on the continuum of care, as well as on the different stages of this continuum, have been weighted as follows.

Overall modeling was performed on the non-continuum of maternal care. In addition to modeling the overall non-continuum, we also performed modeling for each stage of the continuum (“Non-continuum of maternal care from ANC 1 to ANC 4+” and “Non-continuum of maternal care from ANC 4 + to delivery assisted by skilled health personnel”).

Covariates for logistic regression were selected based on a *p*-value of less than or equal to 0.20 in bivariate analysis. We adjusted for multiple variables simultaneously in the models to ensure the validity of the observed associations.

## 3 Results

### 3.1 Sociodemographic characteristics of participants

The investigators received a response from a total of 5,335 women who had completed the provided questionnaire, resulting in a response rate of 98.79%. It was observed that the median age of the surveyed women was 29 years with an interquartile range (IQR) of 24–36. The majority of the surveyed women, accounting for 91.6%, were married. Additionally, it was noted that 94.5% of the women surveyed were Muslim, while 49.7% belonged to the Fulani tribe. In terms of the women's educational background, 46.3% of the surveyed women had not received any formal education (Table 1).

**Table 1:** Sociodemographic characteristics of 5,335 women who gave birth in the last 12 months before the 2022 survey in Conakry, Guinea.

Variables	Number (%)	Continuum of maternal health care	
		No (%)	Yes (%)
Survey communes			
Dixinn	997 (18.69)	71.70	28.30
Kaloum	896 (16.79)	73.90	26.10
Matoto	1,085 (20.34)	75.60	24.40
Ratoma	1,336 (25.04)	76.70	23.30
Matam	1,021 (19.14)	69.90	30.10
Median age	29 (24, 36)		
Age groups			
25–49 years old	3,204 (60.1)	77.87	22.13
15–24 years old	2,131 (39.9)	67.40	32.60
Educational level			
Not attended school	2,472 (46.3)	83.40	16.60
Attended school	2,863 (53.7)	59.70	40.30
Job			
Unemployed	2,714 (50.9)	84.90	15.10
Employed	2,615 (49.0)	54.80	45.20
Not registered	6		
Marital status			
Married	4,886 (91.6)	72.8	27.2
Unmarried	449 (8.4)	74.4	25.6
Ethnic group			
Soussou	1,597 (29.9)	72.50	27.50
Malinke	725 (13.6)	74.20	25.80
Other	54 (1.0)	72.60	27.40
Fulani	2,649 (49.7)	75.40	24.60
Forester	310 (5.8)	69.90	30.10
Religion			
Muslim	5,041 (94.5)	74.10	25.90
Christian	294 (5.5)	70.68	29.32

### 3.2 Obstetric history and use of antenatal and postnatal visits

This study found that the majority (76.6%) of surveyed women were multiparous, with 50% having two or more living children. The report also revealed that 89.6% of women attended their first antenatal visit, while only 55.4% attended the fourth. During the initial antenatal visit, 85.4% and 80.7% of women respectively received sulfadoxine-pyrimethamine and mosquito nets free of charge. Regarding childbirth, 91.2% of women gave birth in a healthcare facility, with a cesarean section rate of 15.1%. The study also observed that 64.4% of women delivered under the care of qualified personnel, with 58.1% receiving postnatal care within the recommended timeframe. Additionally, it was noted that 70.06% of the 922 women who did not receive insecticide-treated bed nets during the first antenatal care (ANC1) had no education, which constituted 12.11% of the total study sample (Table 2).

**Table 2:** Obstetric history and utilization of antenatal and postnatal visits among 5,335 women who gave birth in the last 12 months preceding the 2022 survey in Conakry, Guinea.

Variables	Number (%)	Continuum of maternal health care	
		No (%)	Yes (%)
Parity			
Multiparous	4,085 (76.6)	73.2	26.8
Primiparous	1,250 (23.4)	72.8	27.20
Children born alive			
>2 children	2,666 (50.0)	73.2	27.2
1 to 2 children	2,664 (49.9)	73.5	26.5
Not registered	5		
Stillborn			
>2 children	31 (0.6)	73.6	26.4
1 to 2 children	5,302 (99.4)	73.02	26.98
Not registered	2		
Children alive			
>2 children	2,599 (48.7)	84.50	15.50
1 to 2 children	2,733 (51.2)	68.80	31.20
Not registered	3		
Antenatal care			
ANC1	4,781 (89.6)		
ANC2	4,648 (87.1)		
ANC3	3,273 (61.3)		
ANC4	2,958 (55.4)		
ANC 5 and above	2,492 (46.7)		
SP/Fansidar at ANC1			
Yes	4,083 (85.4)	73.69	26.31
No	695 (14.5)	72.39	27.61
vNot registered	3		
Reception of LLIN at ANC1			
No	922 (19.28)	77.30	22.70
Yes	3,857 (80.67)	68.80	31.20
Not registered	2		
Combination of education and bed net reception			
Non-educated women who have not received LLIM at ANC 1	646 (12.11)	85.33	14.67
Other women	4,689 (87.89)	44.60	55.40
Place of delivery			
No Health facility	467 (8.8)	73.4	26.6
Health facility	4,868 (91.2)	72.6	27.4
Cesarean delivery			
Yes	805 (15.1)	74,8	25,2
No	4,527 (84.9)	72,99	27,01
Not registered	3		
Assisted Childbirth			
No	1,899 (35.6)		
Yes	3,436 (64.4)		
Postnatal care			
No	2,238 (41.9)		
Yes	3,097 (58.1)		

ANC, antenatal care; SP, sulfadoxine and pyrimethamine; LLIN, long-lasting insecticidal net.

### 3.3 Reception of LLINs at ANC1

The distribution of insecticide-treated bed nets is one of the ways to encourage pregnant women to attend antenatal visits. We hypothesized that the provision of nets to women at the first ANC would act as an incentive to continue maternal health care. This is because of Guinea's poverty challenge. In the course of this study, we observed that 19.28% of women who gave birth in the last 12 months before the survey did not receive insecticide-treated bed nets free of charge during their initial antenatal visits. This proportion varies based on sociodemographic characteristics and obstetric history. It is 13.18% among women aged 25–49, compared to 25.37% among women aged 15–24. Likewise, it is 8.45% among educated women, while it reaches 30.11% among non-educated women. Regarding the “employment” variable, we found that the proportion of women not receiving bed nets during their initial antenatal visits was 6.11% among those employed, compared to 32.46% among those unemployed (Table 3).

**Table 3:** Reception of LLINs at ANC1 based on the characteristics of 5,333 women who gave birth in the last 12 months before the 2022 survey in Conakry, Guinea.

Variables	Reception of LLINs at ANC1	
	Yes *n* (%)	No *n* (%)
Survey communes		
Dixinn	788 (78.99)	209 (21.01)
Matam	836 (81.90)	185 (18.10)
Kaloum	752 (83.88)	144 (16.12)
Matoto	851 (78.39)	234 (21.61)
Ratoma	1,075 (80.44)	261 (19.56)
Age groups		
25–49 years old	2,782 (86.82)	422 (13.18)
15–24 years old	1,590 (74.63)	541 (25.37)
Educational level		
Not attended school	1,728 (69.89)	744 (30.11)
Attended school	2,621 (91.55)	242 (8.45)
Job		
Unemployed	1,833 (67.54)	881 (32.46)
Employed	2,455 (93.89)	160 (6.11)
Marital status		
Married	3,903 (79.88)	983 (20.12)
Unmarried	366 (81.58)	83 (18.42)
Ethnic group		
Soussou	1,354 (84.80)	243 (15.20)
Malinke	571 (78.75)	154 (21.25)
Other	44 (81.52)	10 (18.48)
Fulani	2,165 (81.74)	484 (18.26)
Forester	238 (76.82)	72 (23.18)
Religion		
Muslim	4,120 (81.73)	921 (18.27)
Christian	234 (79.71)	60 (20.29)
Parity		
Multiparous	3,303 (80.86)	782 (19.14)
Primiparous	1,007 (80.57)	243 (19.43)
Children born alive		
>2 children	2,189 (82.12)	477 (17.88)
1 to 2 children	2,114 (79.34)	550 (20.66)
Stillborn		
>2 children	26 (83.63)	5 (16.37)
1 to 2 children	4,124 (77.79)	1,178 (22.21)
Children alive		
>2 children	2,120 (81.56)	479 (18.44)
1 to 2 children	2,184(79.90)	584(20.10)

AN, antenatal care; LLIN, long-lasting insecticidal net.

Our research has revealed that distributing insecticide-treated bed nets can effectively encourage pregnant women to attend antenatal appointments. However, we also discovered that nearly one-fifth (19.28%) of women who gave birth within the past year did not receive these bed nets at no cost during their initial antenatal visits. This percentage varied depending on sociodemographic factors and obstetric history. Notably, the percentage was lower among women aged 25–49 (13.18%) than those aged 15–24 (25.37%), as well as among educated women (8.45%) compared to non-educated women (30.11%). Additionally, our findings showed that employed women were significantly less likely to have missed out on bed nets during their initial antenatal visits (6.11%) compared to unemployed women (32.46%).

### 3.4 Continuum and proportions of loss in maternal healthcare continuum

**Figure 1:**
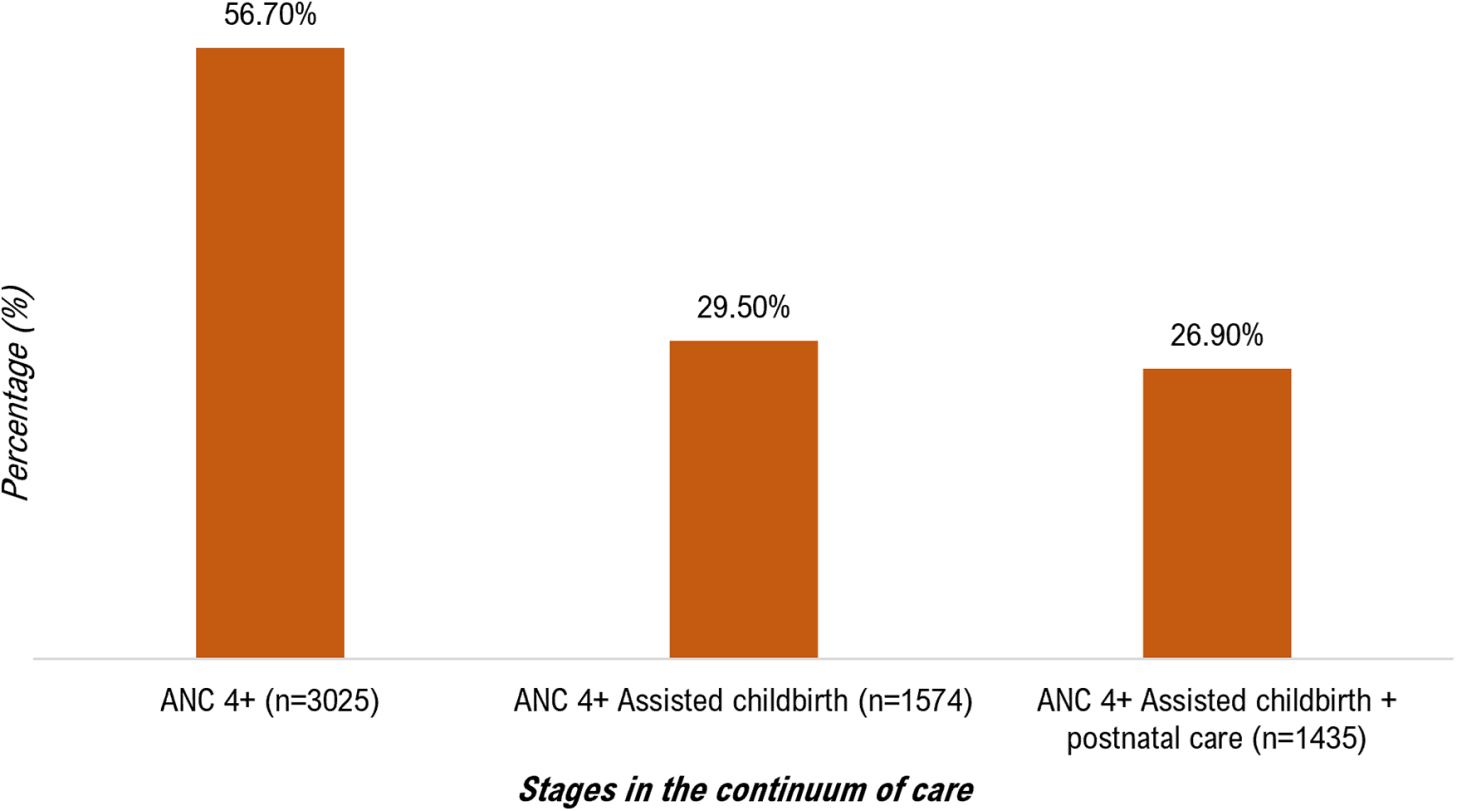
Stages of the continuum of maternal healthcare among 5,335 women who gave birth in the last 12 months before the 2022 survey in Conakry, Guinea.

**Figure 2:**
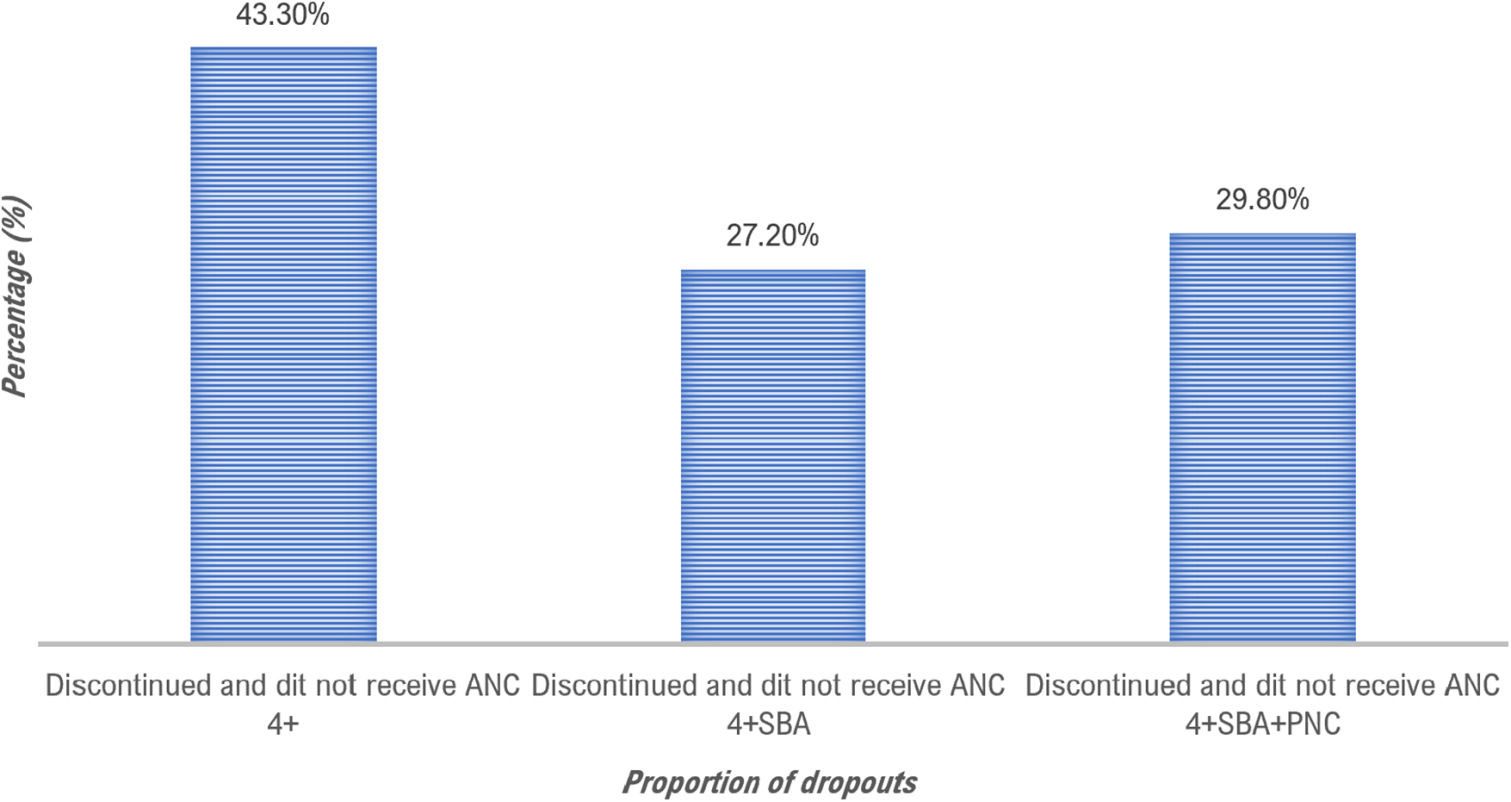
Proportion of losses in the continuum of maternal healthcare among 5,335 women who gave birth in the last 12 months before the 2022 survey in Conakry, Guinea.

This study showed that the (weighted) proportion of women who had completed the various stages of the health care continuum, i.e., the proportion of women who had completed the four prenatal visits, given birth under the care of qualified personnel and received postnatal care within 42 days of delivery” was 26.90% (IC 95%: 22.4–31.3), while 73.1% of women had not completed the various stages of the continuum. Among the 5,335 women surveyed, 56.70% had completed all four prenatal visits (*n* = 2,955). Still using the same denominator (*n* = 5,355), we found that 29.50% of the women surveyed had completed the 4 prenatal visits and had given birth in the hands of qualified health personnel. Finally, using the same denominator (5,355), we found that only 26.90% of women had completed the different stages of the maternal health care continuum (attended the 4 prenatal visits, gave birth in the hands of a professional qualified and had received postnatal care within 42 days of delivery (Figure 1). This last proportion represents the proportion of women who have completed the different stages of the care continuum d (as shown in Figure 1). The study highlights that many women discontinued their care during the postnatal period (29.80%) and at the time of delivery (27.20%), indicating discontinuity in maternal healthcare services (Figure 2).

### 3.5 Factors associated with non-continuum

In our multivariable logistic regression, we found that several factors were associated with the non—continuum of maternal health care (non—completion of the various stages of the continuum by women). These factors include: not having an education compared to having an education (AOR 1.712: 1.456–2.185); being unemployed compared to being employed (AOR 4.232: 3.886–5.562); having two or more living children compared to having one to two living children (AOR 1.198: 1.081–1.453); not receiving a free Long-Lasting Insecticidal Net during the first antenatal care (AOR 2.117: 1.012–2.345); and a combination of not having an education and not receiving LLIM at ANC1 (AOR 6.216: 4.341–8.543) (Table 4).

**Table 4:** Factors associated with non-continuum of maternal healthcare services among 5,335 women who gave birth in the last 12 months before the 2022 survey in Conakry, Guinea.

Variables	Continuum of maternal Health care in weighted value (*n* = 5,335)		Multivariate analysis of the non-continuum of maternal care								
			Non-continuum of maternal Health care from ANC 1 to ANC 4+ (*n* = 2,955)			Non-continuum of maternal health care from ANC4 + to Assisted Childbirth (*n* = 1,462)			Non-Continuum of maternal Health care (ANC4 + Assisted childbirth + postnatal care) = global non-continuum of maternal Health care (*n* = 1,302)		
	No (%)	Yes (%)	AOR	95% CI	*p*-value	AOR	95% CI	*p*-value	AOR	95% CI	*p*-value
Survey communes											
Dixinn	71.70	28.30	0.981	(0.752–1.021)	0.421	1.064	(0.97–1.240)	0.061			
Kaloum	73.90	26.10	1.060	(0.901–1.231)	0.073	1.072	(0.99–1.310)	0.081			
Matoto	75.60	24.40	1.101	(0.672–1.302)	0.069	1.231	(1.106–1.431)	0.003			
Ratoma	76.70	23.30	1.450	(1.246–2.061)	0.006	1.651	(1.321–1.125)	0.002			
Matam	69.90	30.10	1			1					
Age groups											
25–49 years old	77.87	22.13	1.224	(1.098–1.712)	0.004	1.122	(1.043–1.69)	0.008	1.24	(1.041–1.63)	0.007
15–24 years old	67.40	32.60	1			1			1		
Ethnic group											
Soussou	72.50	27.50	1.046	(0.971–1.123)	0.235	1.086	(0.652–1.212)	0.235			
Malinke	74.20	25.80	0.871	(0.076–0.982)	0.321	0.972	(0.064–1.021)	0.453			
Other	72.60	27.40	1.225	(1.086–1.367)	0.009	1.0761	(0.954–1.211)	0.067			
Fulani	75.40	24.60	0.982	(0.543–1.157)	0.561	0.773	(0.651–1.235)	0.081			
Forester	69.90	30.10	1			1					
Religion											
Muslim	74.10	25.90	1.121	(0.965–1.231)	0.432	1.250	(0.991–1.311)	0.421			
Christian	70.68	29.32	1			1					
Educational level											
Not attended school	83.40	16.60	1.842	(1.541–1.762)	<0.001	1.657	(1.5431–2.451)	<0.001	1.712	(1.456–2.185)	<0.001
Attended school	59.70	40.30	1			1			1		
Job											
Unemployed	84.90	15.10	3.65	(3.210–5.231)	<0.001	4.561	(3.543–612)	<0.001	4. 232	(3.886–5.562)	<0.001
Employed	54.80	45.20	1			1			1		
Children alive											
>2 children	84.50	15.50	1.218	(1.0981–1.561)	0.001	1.177	(1.086–1.361)	0.001	1.198	(1.081–1.453)	0.001
1 to 2 children	68.80	31.20	1			1			1		
Reception of LLIN at ANC 1											
No	77.30	22.70	2.353	(1.010–2.216)	0.030	2.101	(1.03–2.123)	0.041	2.117	(1.012–2.345)	0.032
Yes	68.80	31.20	1			1			1		
Combination of education and bed net reception											
Non-educated women who have not received LLIM at ANC 1	85.33	14.67	5.651	(3.546–7.651)	<0.001	7.023	(5.651–8.892)	<0.001	6.216	(4.341–8.543)	<0.001
Other women	44.60	55.40	1			1			1		
SP/Fansidar at ANC 1											
Yes	77.70	22.30	1.231	(1.120–1.651)	0.0024	1.103	(0.876–1.452)	0.354	1.114	(0.930–1.335)	0.240
No	67.60	32.40	1			1			1		

SP, sulfadoxine and pyrimethamine; ANC, antenatal care; LLIN, long-lasting insecticidal net, AOR, adjusted odds ratio; DK, don't know; CI, confidence interval.

## 4 Discussion

This study was conducted to estimate the proportion of women who had completed the various stages of the continuum of maternal health care, i.e., those who had completed the four antenatal visits, given birth under the care of qualified personnel, and received postnatal care within 42 days of delivery, during the 12 months preceding the survey in Conakry, as well as to identify the factors associated with non-completion of the various stages of the continuum of maternal care. A total of 5,335 women were interviewed during the study, and the results indicate that only 26.90% had completed the various stages of the maternal care continuum, while 73.1% had not. These statistics are better than those found by Camara BS et al. in Guinea in 2018, where only 20% of women had completed the various stages of the maternal healthcare continuum compared with 80% of non-completion of the various stages. The findings of this study hold significant implications for maternal healthcare policies and programs in Guinea and other countries with similar contexts (14). Based on these results, we can conclude that the level of non-continuum of maternal health care has decreased slightly, from 80% in 2018 to 73.1% in 2022. Despite this improvement, the result is still far from the national target. Guinea's 2015–2024 National Health Development Plan aims to achieve a continuum level of 73% against a non-continuum level of 27% by 2022 (16).

Several studies carried out in different regions have produced similar results regarding the continuum of maternal healthcare. In particular, a study carried out in Pakistan in 2021 (22) and another in Ethiopia in 2020 (12) determined that the probabilities of women completing the various stages of the continuum of care were 22.3% vs. 77.7% non-completion and 21.6% vs. 78.4%, respectively. In contrast, Charlotte et al. conducted a study in Benin in 2022 and obtained higher results, with 30% of women having completed the various stages of the continuum of care, compared with 70% who had not (21, 23).

The variations in estimates of the level of maternal healthcare continuum observed in the aforementioned studies could be attributed to differences in sample size and sociocultural variations. Another possible explanation could be the study period; in our study, we utilized a recall period of 12 months, whereas other studies used a recall period of 5 years. The use of a longer study period to retrospectively evaluate the utilization of maternal healthcare services before the survey could potentially increase recall bias among the women involved.

There are several reasons why certain women may not receive the appropriate maternal healthcare services they require. One of these reasons is the lack of access to healthcare services, which can be dependent on a number of factors. These may include widely held beliefs or traditional practices, as well as financial constraints that can make it difficult for women to pay for consultations, standard delivery fees, or care in private facilities for ultrasounds.

Our study showed that multiple factors contribute to non-completion of the different stages of the maternal care continuum. Specifically, women with no education are 1.83 times more likely (or 83% more likely) to fail to complete the various stages of the maternal care continuum than other women with some level of education. This finding highlights the negative impact that a lack of education can have on healthcare utilization. It's logical that the less educated a woman is, the less likely she is to take advantage of healthcare services. A study conducted by Atnafu et al. in 2020 in Ethiopia confirms this conclusion. This study revealed that women who could read and write were 2.70 times more likely to benefit from the full range of maternal health services than those who could not read and write (12).

Our findings suggest that the low level of education among young girls in Guinea (13); may explain our results. Education can improve women's knowledge, access to information, and ability to understand advocacy messages through media and healthcare providers. Additionally, women with more education may have greater awareness of the maternal and child health services that are exempt.

This study also revealed that unemployment seems to be a significant factor associated with discontinuous maternal healthcare. In your sample, women without formal employment are 4.2 times more likely not to complete the various stages of the maternal care continuum than women with formal employment. This indicates that employment status may influence access to and the continuity of maternal healthcare. A study by Tesfa et al. in Ethiopia in 2022 also found that unemployment was significantly associated with incomplete use of maternal healthcare services (not continuum) (24). Women without employment may be preoccupied with the daily struggle for survival through informal activities, making it difficult for them to prioritize healthcare service utilization. This information suggests designing specific interventions aimed at improving access to and continuity of maternal healthcare among unemployed mothers. This result underlines the importance of considering socio-economic conditions in public health strategies aimed at improving maternal health.

In addition, the study showed that the number of children is a factor significantly associated with interruption of the maternal health care continuum. Mothers with two or more living children were 1.198 times more likely (or 20% more likely) not to complete the various stages of the maternal health care continuum than those with fewer than two children. Women with multiple children may prioritize their childbirth experience over the completeness of maternal healthcare services. It is therefore essential to offer education and information sessions to women during antenatal care visits, whatever their previous childbirth experience, in order to improve the continuum of maternal health care aimed at reducing maternal and neonatal deaths.

Finally, mothers who did not receive an insecticide-treated bed net during their initial prenatal visits are 2.117 times more likely to not complete the various stages of the maternal healthcare continuum compared to those who did receive one. This result shows that the lack of encouragement for women during maternal healthcare consultations could also limit women's use of healthcare services. This is the case for women in this study who did not receive the bed net during their first antenatal visits. The above result suggests that the distribution of mosquito nets encourages pregnant women to attend regular prenatal consultations, which are essential for screening, prevention of complications, health education and fetal monitoring. Greater participation improves maternal and neonatal health outcomes. Incorporating this distribution reinforces the integrated approach to care, showing that prenatal services can address multiple needs simultaneously, increasing confidence and adherence to ongoing care. In summary, this holistic strategy improves malaria prevention, antenatal care uptake, confidence in the health system and maternal and child health outcomes, strengthening the continuum of care.

The various results related to factors associated with the non-continuum of maternal healthcare highlight how sociodemographic characteristics and other factors like the organization of healthcare services can influence the continuum of maternal healthcare. After combining the variables “education and the distribution of insecticide-treated bed nets during ANC,” we observed a significant increase in the likelihood of not completing maternal care. Thus, the chance of not completing maternal health care was 6.216 times higher among uneducated mothers who did not receive insecticide-treated nets during ANC1 than among those who did. This result underscores how the lack of incentive for non-educated women can exacerbate the low utilization of maternal and child health services, highlighting the need for the country to develop and implement health promotion initiatives.

### 4.1 Strengths and limitations of the study

This study provides new data on the continuum of maternal healthcare in Conakry, Guinea. The sample size of interviewed women was sufficient. However, waiting five years between each demographic and health survey to assess performance and make necessary adjustments seems too long for a healthcare system that aims to reduce maternal and infant mortality efficiently. Many gaps could persist, and they might only become evident after five years. To our knowledge, our study is the first to present results on the continuum of maternal healthcare outside the typical cycle of demographic and health surveys in Guinea. Thus, the findings of this study can help decision-makers in the Guinean health department adjust interventions for women and children while awaiting a new demographic and health survey.

One possible limitation of this study is social desirability bias, as the interviews were conducted by medical students at the end of their training cycle. To mitigate this, we encouraged women to feel comfortable and tell the truth. Also, to reduce desirability bias, we took gender into account in forming the data collection teams. Each team consisted of one woman and one man. In certain instances, when the interviewed women preferred, only the female interviewer conducted the interview. Such cases were rare in this study (less than 1% of the sample). Another potential bias could result from women's recall due to the extended duration of the study. Women might have difficulty remembering the services they received during their previous obstetric visits, leading to overestimations or underestimations of the level of care. We minimized this issue by teaching women recall techniques repeatedly.

Another limitation of this study is that it only collected data from Conakry. Considering the prefectures in the interior of the country could change the estimation of the level of continuum. However, given that the level of continuum is low in Conakry (where access to healthcare services is better), the situation is probably even more concerning in the interior of the country, especially in rural areas. This leads us to claim that the completeness of maternal healthcare remains a significant health problem in Guinea.

Furthermore, since this study is cross-sectional, it does not allow for the examination of causality between the studied independent variables and the non-continuum of maternal healthcare. Additionally, we did not explore women's satisfaction with maternal healthcare services received during pregnancy, childbirth, and postpartum. A study exploring customer satisfaction and the quality of maternal and child healthcare services would be interesting to better understand the challenges related to the continuum of maternal healthcare.

## 5 Conclusion

According to a recent study, only 26.90% of women who had given birth in the 12 months prior to the survey had completed the various stages of the maternal health continuum, while 73.1% had not. These figures are well below the national target of 73% completion, which is essential to reduce maternal and infant morbidity and mortality rates. The study found that several factors, including not attending school, being unemployed, having two or more living children, and not receiving a bed net during the first antenatal visit, were associated with the non-continuity of maternal healthcare. To address these issues, significant additional efforts are needed in Guinea, particularly in improving maternal healthcare continuity. This can be achieved by strengthening education and information for pregnant women during early antenatal visits through interpersonal communication, implementing financial support measures for certain women, digitizing the antenatal visits registry, and creating an appointment reminder system. These measures can help reduce losses between the first and fourth antenatal visits and increase the proportion of women returning to healthcare facilities for postpartum care within the required timeframe. Moreover, improving the availability of quality human resources such as doctors, nurses, and midwives at peripheral healthcare facilities such as health centers and health posts, and increasing healthcare coverage by establishing new health centers and promoting the private sector, could enhance the proportion of women delivering under the care of qualified personnel.

## Data Availability

The raw data supporting the conclusions of this article will be made available by the authors, without undue reservation.

## References

[1] Alkema L Zhang S Chou D Gemmill A Moller AB Fat DM A Bayesian approach to the global estimation of maternal mortality. Ann Appl Stat. (2017) 11(3):1245–74. 10.1214/16-AOAS1014

[2] World Health Organization. Trends in Maternal Mortality 2000 to 2017: Estimates by wHO, UNICEF, UNFPA, World Bank Group and the United Nations Population Division: Executive Summary. Geneva, Switzerland: World Health Organization (2019). Available online at: https://apps.who.int/iris/handle/10665/327596

[3] World Health Organization. Maternal mortality. (2019). Available online at: https://www.who.int/news-room/fact-sheets/detail/maternal-mortality (Accessed October 12, 2022).

[4] Say L Chou D Gemmill A Tunçalp Ö Moller AB Daniels J Global causes of maternal death: a WHO systematic analysis. Lancet Glob Health. (2014) 2(6):e323–33. 10.1016/S2214-109X(14)70227-X 25103301

[5] United Nations. The Millennium Development Goals Report 2015 | United Nations Development Programme [Internet]. UNDP. Available online at: https://www.undp.org/publications/millennium-development-goals-report-2015 (Accessed November 25, 2022).

[6] Haile D Kondale M Andarge E Tunje A Fikadu T Boti N. Level of completion along continuum of care for maternal and newborn health services and factors associated with it among women in Arba Minch Zuria Woreda, Gamo Zone, Southern Ethiopia: a community based cross-sectional study. PloS One. (2020) 15(6):e0221670. 10.1371/journal.pone.0221670 32511230 PMC7279583

[7] World Health Organization. Health in 2015: from MDGs to SDGs—World | ReliefWeb. Available online at: https://reliefweb.int/report/world/health-2015-mdgs-sdgs (Accessed November 25, 2022).

[8] World Health Organization. The World Health Report 2005. Make every mother and child count. (2005). Available online at: https://www.who.int/publications-detail-redirect/9241562900 (accessed November 25, 2022).

[9] Iqbal S Maqsood S Zakar R Zakar MZ Fischer F. Continuum of care in maternal, newborn and child health in Pakistan: analysis of trends and determinants from 2006 to 2012. BMC Health Serv Res. (2017) 17(1):189. 10.1186/s12913-017-2111-9 28279186 PMC5345258

[10] Mothupi MC Knight L Tabana H. Measurement approaches in continuum of care for maternal health: a critical interpretive synthesis of evidence from LMICs and its implications for the South African context. BMC Health Serv Res. (2018) 18(1):539. 10.1186/s12913-018-3278-4 29996924 PMC6042348

[11] Yeji F Shibanuma A Oduro A Debpuur C Kikuchi K Owusu-Agei S Continuum of care in a maternal, newborn and child health program in Ghana: low completion rate and multiple obstacle factors. PloS One. (2015) 10(12):e0142849. 10.1371/journal.pone.0142849 26650388 PMC4674150

[12] Atnafu A Kebede A Misganaw B Teshome DF Biks GA Demissie GD Determinants of the Continuum of maternal healthcare services in Northwest Ethiopia: findings from the primary health care project. J Pregnancy. (2020) 2020:1–8. 10.1155/2020/4318197 PMC7471826 32908704

[13] Institut National de la Statistique du Ministère du Plan et du Développement Economique de Guinée. Enquête de Démographie et de Santé de Guinée, 2018. (2019).

[14] Camara BS Benova L Delvaux T Sidibé S Marie El Ayadi A Peeters Grietens K Women’s progression through the maternal continuum of care in Guinea: evidence from the 2018 Guinean demographic and health survey. Trop Med Int Health. (2021) 26(11):1446–61. 10.1111/tmi.13661 34310807 PMC9292596

[15] Institut National de la Statistique de la Guinée. Des statistiques fiables pour la prise de décision. Available online at: https://www.stat-guinee.org/ (Accessed November 30, 2022).

[16] Ministère de la Santé. Plan National de Développement Sanitaire (PNDS) 2015–2024 de la République de Guinée. (2015)).

[17] Institut National de la Statistique, Ministère du Plan et de la Coopération. Recensement Général de la Population et de l’habitat—3 (RGPH-3), (2014). Guinée).

[18] Organisation Mondiale de la Santé (OMS). Enquête de couverture vaccinale par sondage en grappes: manuel de référence. Available online at: https://cdn.who.int/media/docs/default-source/immunization/immunization-coverage/vaccination_coverage_cluster_survey_fr.pdf (Accessed November 26, 2022).

[19] World Health Organization. Making Pregnancy Safer Department of Reproductive Health and Research. Geneva: World Health Organization; (2004). Available online at: https://www.who.int/publications/i/item/9241591692

[20] Kikuchi K Okawa S Zamawe COF Shibanuma A Nanishi K Iwamoto A Effectiveness of Continuum of care—linking Pre-pregnancy care and pregnancy care to improve neonatal and perinatal mortality: a systematic review and meta-analysis. Simeoni U, éditeur. PLoS One. (2016) 11(10):e0164965. 10.1371/journal.pone.0164965 27788176 PMC5082954

[21] Owili PO Muga MA Chou YJ Hsu YHE Huang N Chien LY. Associations in the continuum of care for maternal, newborn and child health: a population-based study of 12 sub-saharan Africa countries. BMC Public Health. (2016) 16(1):414. 10.1186/s12889-016-3075-0 27188624 PMC4869316

[22] Humaira M Elizabeth H Catherine B. Factors affecting rural women’s utilisation of continuum of care services in remote or isolated villages or Pakistan—a mixed-methods study. ScienceDirect. Volume. (2021) 34(3):257–65. 10.1016/j.wombi.2020.04.001 32360107

[23] Gryseels C Dossou J Vigan A Boyi Hounsou C Kanhonou L Benova L Where and why do we lose women from the continuum of care in maternal health? A mixed-methods study in Southern Benin. Trop Med Int Health. (2022) 27(3):236–43. 10.1111/tmi.13729 35098607 PMC9306704

[24] Alamneh TS Teshale AB Yeshaw Y Alem AZ Ayalew HG Liyew AM Barriers for health care access affects maternal continuum of care utilization in Ethiopia; spatial analysis and generalized estimating equation. PLoS One. (2022) 17(4):e0266490. 10.1371/journal.pone.0266490 35452475 PMC9032438

